# Assessment of chemotherapy resistance changes in human colorectal cancer xenografts in rats based on MRI histogram features

**DOI:** 10.3389/fonc.2024.1301649

**Published:** 2024-01-31

**Authors:** Min-Yi Wu, Qi-Jia Han, Zhu Ai, Yu-Ying Liang, Hao-Wen Yan, Qi Xie, Zhi-Ming Xiang

**Affiliations:** ^1^ Department of Radiology, Guangzhou Panyu Central Hospital, Guangzhou, China; ^2^ Department of Radiology, Guangzhou First People’s Hospital/Department of Medical Imaging, Nansha Hospital, Guangzhou, Guangzhou, China

**Keywords:** human colorectal cancer xenografts, rats, MRI histogram features, chemotherapy, Resistance

## Abstract

**Purpose:**

We investigated the value of magnetic resonance imaging (MRI) histogram features, a non-invasive method, in assessing the changes in chemoresistance of colorectal cancer xenografts in rats.

**Methods:**

A total of 50 tumor-bearing mice with colorectal cancer were randomly divided into two groups: control group and 5-fluorouracil (5-FU) group. The MRI histogram characteristics and the expression levels of p53 protein and MRP1 were obtained at 24 h, 48 h, 72 h, 120 h, and 168 h after treatment.

**Results:**

Sixty highly repeatable MRI histogram features were obtained. There were 16 MRI histogram parameters and MRP1 resistance protein differences between groups. At 24 h after treatment, the MRI histogram texture parameters of T2-weighted imaging (T2WI) images (10%, 90%, median, energy, and RootMeanSquared) and D images (10% and Range) were positively correlated with MRP1 (r = 0.925, p = 0.005). At 48 h after treatment, histogram texture parameters of apparent diffusion coefficient (ADC) images (Energy) were positively correlated with the presence of MRP1 resistance protein (r = 0.900, p = 0.037). There was no statistically significant difference between MRI histogram features and p53 protein expression level.

**Conclusions:**

MRI histogram texture parameters based on T2WI, D, and ADC maps can help to predict the change of 5-FU resistance in colorectal cancer in the early stage and provide important reference significance for clinical treatment.

## 1 Introduction

Colorectal cancer (CRC) is one of the most common malignant tumors in humans (1–3). 5-Fluorouracil (5-FU)-based chemotherapy palliative treatment has become an important means of treatment. However, the tumor cells may develop drug resistance quickly and easily in the course of chemotherapy, making the progression-free survival of patients only 8.7–12.3 months (4, 5). Clinicians need to adjust treatment regimens based on tumor drug resistance, which is an important step in guiding treatment. Currently, the detection of chemotherapy resistance in tumors is mainly performed through invasive laboratory tests, such as biopsy or postoperative pathology. However, due to the differences in sampling sites, the samples cannot fully reflect the drug resistance of tumor cells and are not always well accepted by patients (6). Therefore, it is urgent to find a relatively accurate and comprehensive non-invasive method for drug resistance assessment of colorectal cancer. The nude mouse model of colorectal cancer transplantation can well reflect the pathological characteristics of human colorectal cancer, and it is well controlled in the experiment (7).

The magnetic resonance imaging (MRI) histogram feature is becoming more and more popular among scholars (8, 9). It plays an important role in predicting the outcome of colorectal cancer treatment. First, it can detect tissue changes that are not easily detected by the naked eye by quantifying the relationship between gray patterns and pixels in the image, thus making up for the deficiency of traditional image analysis methods. Second, it can well distinguish subtle differences in texture information of images, evaluate tumor heterogeneity, and carry out differential diagnosis, efficacy evaluation, and prognosis prediction of tumors. You J et al. performed preoperative T staging of rectal cancer based on whole-tumor texture analysis on MRI (10). Li J et al. found that magnetic resonance diffusion-weighted imaging histogram parameters can be used as biomarkers to predict lymph node metastasis in T3 rectal cancer (11). Importantly, it is a non-invasive test that is easily accepted by patients and can be widely used in clinical practice (12, 13). Therefore, the MRI histogram feature may be a good method to detect drug resistance in colorectal cancer.

Multidrug resistance-associated protein 1 (MRP1), a member of multidrug resistance protein MRP, is a glutathione transport pump (GS-X) widely expressed in human tissues. Many studies have found that tumor-associated macrophages (TAMs) in colorectal cancer can confer resistance to 5-FU treatment through MRP1-dependent drug efflux mediated by cell–cell interactions (14). p53 gene is located on the short arm of chromosome 17 (17p13) and encodes nucleic acid phosphoprotein (15). Mutant p53 protein can be detected in more than 50% of human tumors and approximately 70% of colorectal cancer cases (16, 17). It promotes tumor progression and resistance to therapy, and this mutant form has become the most common prognostic indicator of tumor recurrence and death from cancer (18, 19). In this study, we investigated the feasibility of MRI histogram features to predict chemotherapy response in CRC-resistant xenograft mice by analyzing the correlation between MRI histogram features and MRP1 protein and p53 protein expression.

## 2 Materials and methods

### 2.1 Establishment of animal model

This study used male and female BALB/c nude mice (6–8 weeks old, weighing 20–25 g). All the animals were housed in an environment with a temperature of 22°C ± 1°C, relative humidity of 50% ± 1%, and a light/dark cycle of 12/12 h and had free access to standard water and food (SYXK-2016-0168). All procedures were conducted in accordance with the “Guiding Principles for the Care and Use of Animals” (China) and were approved by the Institutional Animal Care and Use Committee of Guangzhou Medical University (GY2017-007).

Colorectal cancer (SW480) tissue was implanted subcutaneously in the back of nude mice to establish a xenograft model (20). All procedures were performed under the induction of chloral hydrate (4.5% chloral hydrate, 2 mL/100 g body weight, i.p.) to minimize pain. Mice were maintained in a specific pathogen-free (SPF) laboratory. When the tumor diameter was approximately 1.5 cm, the mice were randomly divided into two groups: control group and 5-FU group. The dose of 5-FU and normal saline was as follows: 25 mg in tumor diameter of 0.5–0.9 cm, 50 mg in tumor diameter of 1.0–1.4 cm, and 75 mg in tumor diameter above 1.5 cm. The route of administration was direct intra-tumoral injection.

### 2.2 Western blotting analysis

Western blotting analysis was used to detect the protein expression of p53 and MRP1, as described in the manufacturer’s package (Phototope^®^-HRP Western Blot Kit; Cell Signaling Technology, Danvers, MA, USA). Cell extracts were obtained from frozen tumor tissue at −30°C. Immunoblot analysis was performed using anti-p53 monoclonal antibodies (Santa Cruz Biotechnology, Dallas, TX, USA) and MRP1 monoclonal antibodies (Santa Cruz Biotechnology). Subsequent protein detection was performed using an enhanced chemiluminescence (ECL) detection system (Hitachi, Tokyo, Japan).

The band intensities [integrated optical density (IOD)] of protein expression stated above were scanned into the computer and analyzed using Image-Pro Plus 6.0 software. The relative IOD (RIOD) of protein expression was calculated as the IOD of the protein in the control group and therapeutic groups at each time point divided by the corresponding IOD of GAPDH (internal control).

### 2.3 MRI examination

At 24 h, 48 h, 72 h, 120 h, and 168 h after 5-FU treatment, five tumor-bearing mice in each group were randomly selected for MRI examination. MRI scan was performed using Siemens MAGNETOM Verio 3.0T superconducting magnetic resonance imaging system and Shanghai Chenguang 8-channel phased-array animal coil. The MRI sequence included the following: 1) T1-weighted imaging (T1WI) (turbo spin echo (TSE)) sequence, repetition time/echo time (TR/TE) = 2,780 ms/15 ms, slice thickness = 2 mm, interval = 0, field of view (FOV) = 200 mm × 200 mm, Neuroimaging Association Score (NAS)= 4, SENSE acceleration factor = 2, acquisition matrix = 200 × 200, reconstruction matrix = 768 × 768, imaging in axial plane, coronal plane and sagittal plane, imaging time = 2 min 2 s; 2) T2-weighted imaging (T2WI) (TSE) sequence, TR/TE = 4,500 ms/110 ms, slice thickness =2 mm, interval = 0, FOV = 128 mm × 128 mm, NAS = 4, SENSE acceleration factor = 2, acquisition matrix = 128 × 128, reconstruction matrix = 512 × 512, row axis plane, coronal plane, sagittal plane imaging, imaging time = 2 min 2 s; (3) diffusion-weighted imaging (DWI): single-shot echo-planar imaging (EPI) was used, TR/TE = 3,400 ms/60 ms, diffusion sensitive gradient field was applied iso-tropically, b value (50, 100, 150, 200, 400, 800, and 1,200 s/mm^2^), slice thickness 2 mm, interval = 0, FOV = 220 mm × 220 mm, NAS = 3, SENSE acceleration factor = 2, acquisition matrix = 220 × 220, reconstruction matrix = 308 × 308, row axis imaging, imaging time = 13 min 39 s.

### 2.4 Data analysis

After the MRI examination, the original data were analyzed using the picture archiving and communication system (PACS) system. The intravoxel incoherent motion–diffusion-weighted imaging (IVIM-DWI) was imported into the MITK Diffusion on analysis software in Digital Imaging and Communications in Medicine (DICOM) format, and the bi-exponential model parameter map was obtained, including a true diffusion coefficient map (D map), false diffusion coefficient map (D* map), perfusion coefficient map (f map), DWI map, and apparent diffusion coefficient map (ADC map).

### 2.5 MR histogram texture feature acquisition

**Figure 1:**
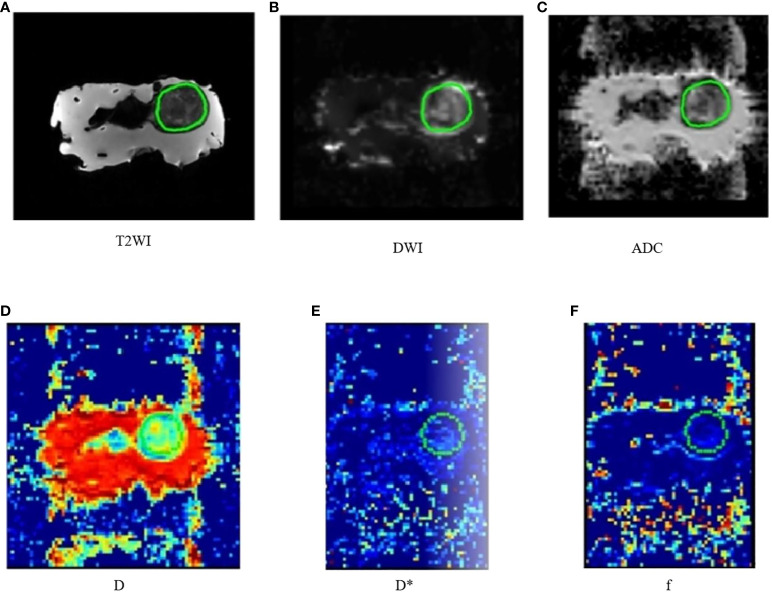
Nude mouse transplanted tumors in different MRI sequences **(A–F)**.

The region of interest (ROI) was delineated by three radiologists. The largest tumor layer was delineated as ROI, along the outer edge of the tumor, and an attempt was made to include the entire tumor range (**Figures 1A–F**). The MRI histogram features of the tumor were obtained using The MR Station software. A total of 108 MRI histogram texture features were obtained.

### 2.6 Statistical analysis

All data were statistically analyzed using Statistical Package for the Social Sciences (SPSS) 26.0 software. The Shapiro–Wilk test was used for normality analysis. The intragroup correlation coefficient was used to detect the repeatability of each MRI histogram texture parameter measurement, and the MRI histogram texture parameter with intraclass correlation coefficient (ICC) ≥0.75 was selected for statistical analysis. t-Test or the Mann–Whiney test was used to compare MRI histogram texture parameters, p53 protein, and MRP1 between the control group and the 5-FU group. Finally, the correlation of MRI histogram texture parameters with the p53 protein and drug resistance protein at five time points after 5-FU administration was analyzed. p < 0.05 was considered statistically significant.

## 3 Results

### 3.1 MRI histogram texture parameters with high repeatability (ICC ≥ 0.75)

**Figure 2:**
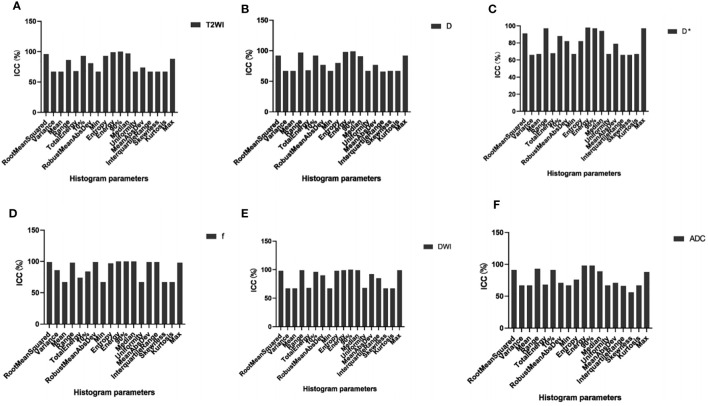
MRI histogram texture parameters(ICC0.75) **(A-F)**. ICC, intraclass correlation coefficient.

Of the 108 MRI histogram texture parameters, 60 had high repeatability and were analyzed. The T2WI maps showed nine MRI histogram texture parameters (ICC = 0.995). The D maps showed 10 histogram parameters (ICC = 0.985). The D* maps showed 10 histogram parameters (ICC = 0.978). The f maps showed 12 histogram parameters (ICC = 0.985). The DWI maps showed 11 histogram parameters (ICC = 0.996). The ADC maps showed eight histogram parameters (ICC = 0.978) (**Figure 2**) (**Table 1**).

**Table 1:** MRI histogram texture parameters with high repeatability [ICC (95%, CI), ICC ≥ 0.75].

Parameters	T2WI	D value	D* value	f value	DWI	ADC
10%	0.928 (0.903, 0.948)	0.921 (0.895, 0.943)	0.875 (0.834, 0.909)	0.835 (0.784, 0.878)	0.957 (0.942, 0.969)	0.907 (0.876, 0.933)
90%	0.995 (0.993, 0.996)	0.985 (0.979, 0.989)	0.973 (0.964, 0.981)	0.996 (0.995, 0.997)	0.996 (0.994, 0.997)	0.978 (0.97, 0.984)
Range	0.86 (0.815, 0.897)	0.965 (0.953, 0.975)	0.969 (0.958, 0.978)	0.975 (0.966, 0.982)	0.988 (0.983, 0.991)	0.925 (0.899, 0.946)
Median	0.969 (0.958, 0.978)	0.907 (0.876, 0.933)	0.938 (0.916, 0.955)	0.995 (0.994, 0.997)	0.988 (0.983, 0.991)	0.894 (0.859, 0.923)
Entropy	0.933 (0.909, 0.951)	0.798 (0.737, 0.85)	0.821 (0.767, 0.868)	0.97 (0.96, 0.979)	0.981 (0.974, 0.986)	0.76 (0.692, 0.82)
Energy	0.991 (0.987, 0.993)	0.979 (0.972, 0.985)	0.978 (0.97, 0.985)	0.996 (0.995, 0.997)	0.99 (0.986, 0.993)	0.978 (0.97, 0.984)
Max	0.882 (0.843, 0.914)	0.916 (0.888, 0.939)	0.971 (0.961, 0.979)	0.975 (0.966, 0.982)	0.987 (0.982, 0.991)	0.883 (0.845, 0.915)
RootMeanSquared	0.957 (0.942, 0.969)	0.922 (0.895, 0.944)	0.905 (0.873, 0.931)	0.991 (0.988, 0.994)	0.978 (0.971, 0.985)	0.905 (0.873, 0.931)
RobustMeanAbsDev	0.806 (0.748, 0.856)	0.766 (0.699, 0.825)	0.824 (0.77, 0.87)	0.989 (0.985, 0.992)	0.895 (0.861, 0.924)	/
MeanAbsDev	/	0.772 (0.707, 0.83)	0.787 (0.725, 0.841)	0.991 (0.987, 0.993)	0.924 (0.899, 0.945)	/
Inter-quartile Range	/	/	/	0.991 (0.988, 0.994)	0.851 (0.804, 0.891)	/
Variance	/	/	/	0.856 (0.811, 0.894)	/	/

CI, confidence intervals; ICC, intraclass correlation coefficient; DWI, diffusion-weighted imaging; ADC, apparent diffusion coefficient.

### 3.2 Difference analysis of the MRI histogram texture parameters between the 5-FU group and control group (**Table 2**)

Between the 5-FU group and the control group, there were differences in the six MRI histogram texture parameters of 10%, 90%, Median, Energy, Robust-Mean-Abs-Dev, and RootMeanSquared in T2WI maps, and the difference in Energy was the most obvious (p = 0.001). There were differences in the four MRI histogram texture parameters of 10%, Range, Entropy, and RootMeanSquared in the D maps, and the difference in Energy was the most obvious (p = 0.002). There were differences in the five MRI histogram texture parameters of 10%, Range, Entropy, Energy, Max, and Interquartile in DWI maps, and the difference in Entropy was the most obvious (p = 0.005). There were differences in energy in the D* maps and ADC maps (p = 0.002 and p = 0.034, respectively) (**Table 2**).

**Table 2:** Difference analysis of MRI histogram texture parameters between 5-FU group and the control group.

Texture parameter	Control group (n = 25)	5-FU group (n = 25)	p-Value
T2WI maps			
10%	531.68 (343.25, 688.50)	380.7 (183.03, 517.68)	0.018
90%	1,249.25 (934.80, 1,482.10)	767.2 (473.4, 1,216.75)	0.017
Median	757.75 (551.38, 1,042.25)	517.25 (308.82, 793.88)	0.042
Energy	3,276,633,351.00 (2,465,654,986.50, 5,990,568,710.00)	824,904,676.8 (3,765,88,326.8, 1,965,739,638)	0.001
RootMeanSquared	893.46 (687.79, 1,205.16)	571.60 (315.82, 891.66)	0.016
D maps			
10%	536.00 (385.34, 698.50)	403.87 (251.60, 530.82)	0.025
Range	858.93 (669.11, 1,151.16)	730.39 (380.32, 928.27)	0.043
Energy	2,443,009,714.00 (1,306,611,654.50, 3,723,268,077.00)	796,897,395.40 (365,132,333.60, 1,555,503,893.50)	0.002
RootMeanSquared	689.12 (585.15, 948.49)	530.04 (331.94, 728.49)	0.018
D* maps			
Energy	6,468,230,884.00 (4,069,730,225.00, 11,894,205,829.00)	3,073,221,269.25 (2,096,691,201.00, 4,492,560,537.37)	0.002
DWI maps			
10%	5.55 (3.46, 12.46)	12.22 (5.36, 19.19)	0.049
Range	133.59 (90.82, 270.71)	236.44 (236.44, 278.11)	0.014
Entropy	1.85 (1.55, 2.05)	2.26 (1.95, 2.93)	0.005
Max	133.59 (94.77, 270.79)	236.59 (155.69, 280.62)	0.014
Interquartile range	36.16 (26.39, 52.53)	47.75 (37.38, 84.25)	0.045
ADC maps			
Energy	56,748,093.17 (12,289,031.74, 76,078,374.66)	19,327,439.51 (8,267,527.21, 43,112,112.68)	0.034

5-FU, 5-fluorouracil; T2WI, T2-weighted imaging; DWI, diffusion-weighted imaging; ADC, apparent diffusion coefficient.

*Mann–Whitney U test; the median (upper and lower quartiles).

### 3.3 Difference analysis of p53 protein and MRP1 resistance protein between the 5-FU group and the control group (**Table 3**)

In this study, the differences in the expression levels of p53 protein and MRP1 between the 5-FU group and the control group were detected. The expression of MRP1 was significantly different between the two groups (p = 0.000). The expression levels of p53 protein were not different between the two groups (**Table 3**).

**Table 3:** Difference analysis of p53 protein and MRP1 resistance protein between 5-FU group and the control group.

Drug-resistant protein	Control group (n = 25)	5-FU group (n = 25)	p-Value
P53	0.22 (0.18, 0.24)	0.20 (0.17, 0.25)	0.785
MRP1	0.65 (0.41, 0.88)	1.09 (0.82, 1.51)	0.000

5-FU, 5-fluorouracil.

*Mann–Whitney U test; the median (upper and lower quartiles).

### 3.4 Correlation analysis of the MRI histogram texture parameters and expression of MRP1 resistance protein at different time points after 5-FU administration

**Figure 3:**
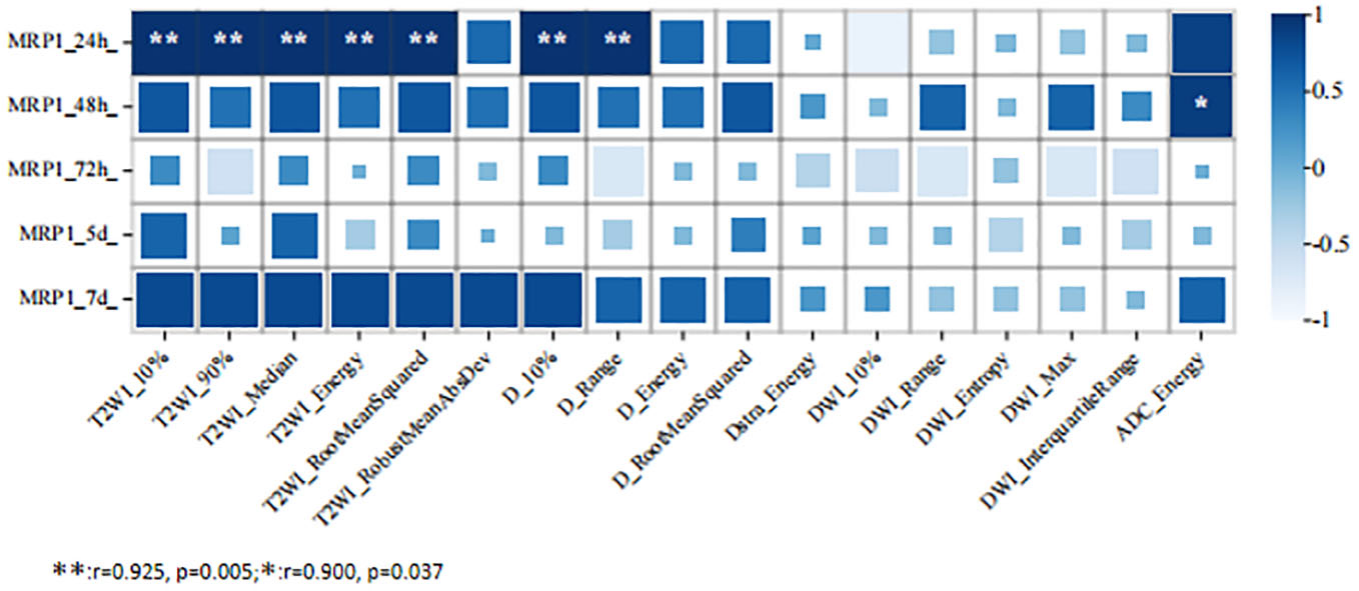
Correlation analysis between MRI histogram texture parameters and MRP1 expression level in 5-fluorouracil (5-FU) group at different time points.

At 24 h after 5-FU administration, there were five MRI histogram texture features (10%, 90%, Median, Energy, and RootMeanSquared) in T2WI maps, and two MRI histogram texture features (10% and Range) in the D maps showed a significant positive correlation with MRP1 (r = 0.975, p = 0.005). At 48 h after 5-FU administration, the MR histogram texture Energy of the ADC map showed a significant positive correlation with MRP1 (r = 0.900, p = 0.03). There was no correlation between the MR texture parameters and the expression of MRP1 at 72 h, 120 h, and 168 h after 5-FU administration (**Figure 3**).

## 4 Discussion

MRP1 can transport chemotherapeutic drugs coupled with reduced glutathione against the concentration gradient and reduce the concentration of chemotherapeutic drugs in tumor cells, thus allowing tumor cells to develop drug resistance. The high expression of MRP1 is one of the important causes of multidrug resistance in colorectal cancer cells. Therefore, we can effectively predict the generation of drug resistance by detecting the expression of MRP1 in tumor cells.

MRI histogram features is a non-invasive imaging technology. It uses mathematical methods to quantify the grayscale, spatial distribution, structure, and other information of MRI image pixels, allowing to extraction of image features of lesions. It accurately reflects the difference in image voxels, evaluates tumor heterogeneity, and performs differential diagnosis, efficacy evaluation, and prognosis prediction of tumors (21, 22). These texture features significantly expand the image information and application value of tumors and have high repeatability (23). Previous studies have also shown that MR histogram features can provide valuable medical imaging information in identifying colorectal cancer malignancy, T staging, and efficacy prediction (24–34).

Based on the above clinical issues and theoretical principles, our study extracted MRI histogram features in MRI multi-parameter imaging (T2WI, DWI, ADC, D, D*, and f) to analyze the relationship between MRI histogram features and the expression levels of MRP1 protein at different time points in human colorectal cancer nude mouse transplanted tumors after 5-FU treatment. The results show that the texture features of the MRI histogram (10%, 90%, Median, Energy, RootMeanSquared, Range, and Energy) are highly correlated with MRP1 expression. They describe the tumor heterogeneity, which reflects high cell density, necrosis, hemorrhage, and myxoid changes within the tumor area. Tumor heterogeneity is an important factor for prognosis. Song et al. found that the texture features of T2WI can provide valuable information for identifying the lymph node infiltration status of rectal cancer (34). In our study, it was found that the MRI histogram features of 10%, 90%, Median, Energy, and RootMeanSquared in T2WI images of colorectal transplanted tumors were highly correlated with MRP1, which could support the study results of Song et al. from another aspect. ADC and D can further reflect tumor tissue proliferation and metabolism, cell density, and blood supply (35–37). In our study, it was found that 10% of D, Range MRI histogram texture features, and Energy MRI histogram texture features in the ADC map were highly correlated with MRP1. However, our study did not find MRI histogram texture features related to MRP1 at 72 h, 120 h, and 168 h after 5-FU treatment. The possible reason for this is that the MRI histogram texture features can only accurately detect changes in drug resistance at a certain point in time, and after the most appropriate detection time, the correlation between the two will be affected by some unknown reasons. This is what we need to find out in our follow-up studies.

In this study, the time points of 24 h, 48 h, 72 h, 120 h, and 168 h after injection were statistically analyzed. The results showed that there was no correlation between MRI histogram texture and p53 protein over time. There were the following two possible reasons: the first possible reason was that histogram texture can only accurately detect changes in drug resistance over a specific time period, and p53 protein expression was not evident in this time period. The second possible reason was that our study was too short and the correlation between the two had not yet been reflected.

## 5 Conclusion

MRI histogram texture can predict the change of drug resistance in colorectal cancer in the early stage. It can provide a reference for the adjustment of therapeutic drugs for patients with colorectal cancer and has positive significance.

## Data availability statement

The original contributions presented in the study are included in the article/supplementary material. Further inquiries can be directed to the corresponding author.

## Ethics statement

Ethical approval was not required for the studies on humans in accordance with the local legislation and institutional requirements because only commercially available established cell lines were used. The animal studies were approved by the Experimental Animal Ethics Committee of Guangzhou Medical College. Experimental Animal Center, Panyu Campus, Guangzhou Medical University, Xinzao Town, Panyu District, Guangzhou. The studies were conducted in accordance with the local legislation and institutional requirements. Written informed consent was obtained from the owners for the participation of their animals in this study.

## Author contributions

M-YW: Writing – original draft, Writing – review & editing. Q-JH: Writing – review & editing. ZA: Writing – original draft. Y-YL: Writing – original draft. H-WY: Writing – review & editing. QX: Writing – review & editing. Z-MX: Writing – review & editing.

## References

[1] Teka MA Yesuf A Hussien FM. Histological characteristics, survival pattern and prognostic determinants among colorectal cancer patients in Ethiopia: A retrospective cohort study. Heliyon (2021) 7(2):e06366. doi: 10.1016/j.heliyon.2021.e06366 33718651 PMC7920880

[2] Siegel RL Miller KD Fedewa SA. Colorectal cancer statistics. CA Cancer J Clin (2017) 67(3):177–93. doi: 10.3322/caac.21395 28248415

[3] Biller LH Schrag D. Diagnosis and treatment of metastatic colorectal cancer: A review. JAMA (2021) 325(7):669–85. doi: 10.1001/jama.2021.0106 33591350

[4] Soerjomataram I Lortet-Tieulent J Parkin DM. Global burden of cancer in 2008: a systematic analysis of disability-adjusted life-years in 12 world regions. Lancet (2012) 380(9856):1840–50. doi: 10.1016/S0140-6736(12)60919-2 23079588

[5] Sun X Hou W Liu X. Targeting REV7 effectively reverses 5-FU and oxaliplatin resistance in colorectal cancer. Cancer Cell Int (2020) 20(1):580. doi: 10.1186/s12935-020-01668-z 33292253 PMC7713438

[6] Cree IA Glaysher S Harvey AL. Efficacy of anti-cancer agents in cell lines versus human primary tumour tissue. Curr Opin Pharmacol (2010) 10(4):375–9. doi: 10.1016/j.coph.2010.05.001 20570561

[7] Hu HF Wang Z Tang WL. Effects of Sophora flavescens aiton and the absorbed bioactive metabolite matrine individually and in combination with 5-fluorouracil on proliferation and apoptosis of gastric cancer cells in nude mice. Front Pharmacol (2022) 13:1047507. doi: 10.3389/fphar.2022.1047507 36438804 PMC9681822

[8] Mannil M Eberhard M von Spiczak J. Artificial intelligence and texture analysis in cardiac imaging. Curr Cardiol Rep (2020) 22(11):131. doi: 10.1007/s11886-020-01402-1 32910325

[9] Delli Pizzi A Chiarelli AM Chiacchiaretta P d'Annibale M Croce P Rosa C. MRI-based clinical-radiomics model predicts tumor response before treatment in locally advanced rectal cancer. Sci Rep (2021) 11(1):5379. doi: 10.1038/s41598-021-84816-3 33686147 PMC7940398

[10] You J Yin J. Performances of whole tumor texture analysis based on MRI: predicting preoperative T stage of rectal carcinomas. Front Oncol (2021) 11:678441. doi: 10.3389/fonc.2021.678441 34414105 PMC8369414

[11] Li J Zhou Y Wang X Yu Y Zhou X Luan K. Histogram analysis of diffusion-weighted magnetic resonance imaging as a biomarker to predict lymph node metastasis in T3 stage rectal carcinoma. Cancer Manag Res (2021) 13:2983–93. doi: 10.2147/CMAR.S298907 PMC8021267 33833581

[12] Xing P Chen L Yang Q. Differentiating prostate cancer from benign prostatic hyperplasia using whole-lesion histogram and texture analysis of diffusion- and T2-weighted imaging. Cancer Imaging (2021) 21(1):54. doi: 10.1186/s40644-021-00423-5 34579789 PMC8477463

[13] Litvin AA Burkin DA Kropinov AA Paramzin FN. Radiomics and digital image texture analysis in oncology (Review). Sovrem Tekhnologii Med (2021) 13(2):97–104. doi: 10.17691/stm2021.13.2.11 34513082 PMC8353717

[14] Zhang L Lu X Xu Y. Tumor-associated macrophages confer colorectal cancer 5-fluorouracil resistance by promoting MRP1 membrane translocation via an intercellular CXCL17/CXCL22-CCR4-ATF6-GRP78 axis. Cell Death Dis (2023) 14(9):582. doi: 10.1038/s41419-023-06108-0 37658050 PMC10474093

[15] Carson DA Lois A. Cancer progression and p53. Lancet (1995) 346(8981):1009–11. doi: 10.1016/S0140-6736(95)91693-8 7475551

[16] Bookstein R Demers W Gregory R. p53 gene therapy in *vivo* of herpatocellular and liver metastatic colorectal cancer. Semin Oncol (1996) 23(1):66–77. 8607033

[17] Liu YY Hill RA Li YT. Ceramide glycosylation catalyzed by glucosylceramide synthase and cancer drug resistance. Adv Cancer Res (2013) 117:59–89. doi: 10.1016/B978-0-12-394274-6.00003-0 23290777 PMC4051614

[18] Grassilli E Narloch R. Inhibition of GSK3B bypass drug resistance of p53-null colon carcinomas by enabling necroptosis in response to chemotherapy. Clin Cancer Res (2013) 19(14):3820–31. doi: 10.1158/1078-0432.CCR-12-3289 23729362

[19] Hodorová I Rybárová S Vecanová J Solár P Plank L Mihalik J. Relation between expression pattern of wild-type p53 and multidrug resistance proteins in human nephroblastomas. Acta histochemica (2013) 115(3):273–8. doi: 10.1016/j.acthis.2012.08.001 22925562

[20] Xie Q Liang BL Wu YH. Synergistic anticancer effect of rAd/P53 combined with 5-fluorouracil or iodized oil in the early therapeutic response of human colon cancer in *vivo* . Gene (2012) 499(2):303–8. doi: 10.1016/j.gene.2012.02.007 22441128

[21] Shi L Zhang Y Nie K. Machine learning for prediction of chemoradiation therapy response in rectal cancer using pre-treatment and mid-radiation multi-parametric MRI. Magn Reson Imaging (2019) 61:33–40. doi: 10.1016/j.mri.2019.05.003 31059768 PMC7709818

[22] Awe AM Rendell VR Lubner MG Winslow ER. Texture analysis: an emerging clinical tool for pancreatic lesions. Pancreas (2020) 49(3):301–12. doi: 10.1097/MPA.0000000000001495 PMC7135958 32168248

[23] Shur J Blackledge M D’Arcy J. MRI texture feature repeatability and image acquisition factor robustness, a phantom study and in silico study. Eur Radiol Exp (2021) 5(1):2. doi: 10.1186/s41747-020-00199-6 33462642 PMC7813908

[24] Zhang B Song L Yin J. Texture analysis of DCE-MRI intratumoral subregions to identify benign and Malignant breast tumors. Front Oncol (2021) 11:688182. doi: 10.3389/fonc.2021.688182 34307153 PMC8299951

[25] MacIver CL Busaidi AA Ganeshan B. Filtration-histogram based magnetic resonance texture analysis (MRTA) for the distinction of primary central nervous system lymphoma and glioblastoma. J Pers Med (2021) 11(9):876. doi: 10.3390/jpm11090876 34575653 PMC8472730

[26] Azoulay A Cros J Vullierme MP de Mestier L Couvelard A Hentic O. Morphological imaging and CT histogram analysis to differentiate pancreatic neuroendocrine tumor grade 3 from neuroendocrine carcinoma. Diagn Interv Imaging (2020) 101(12):821–30. doi: 10.1016/j.diii.2020.06.006 32709455

[27] Sarioglu O Sarioglu FC Akdogan AI. MRI-based texture analysis to differentiate the most common parotid tumours. Clin Radiol (2020) 75(11):877. doi: 10.1016/j.crad.2020.06.018 32703544

[28] Ye R Weng S Li Y. Texture analysis of three-dimensional MRI images may differentiate borderline and Malignant epithelial ovarian tumors. Korean J Radiol (2021) 22(1):106–17. doi: 10.3348/kjr.2020.0121 PMC7772386 32932563

[29] Yin JD Song LR Lu HC. Prediction of different stages of rectal cancer: Texture analysis based on diffusion-weighted images and apparent diffusion coefficient maps. World J Gastroenterol (2020) 26(17):2082–96. doi: 10.3748/wjg.v26.i17.2082 PMC7267694 32536776

[30] Choi TW Kim JH Yu MH Park SJ Han JK. Pancreatic neuroendocrine tumor: prediction of the tumor grade using CT findings and computerized texture analysis. Acta Radiol (2018) 9(4):383–92. doi: 10.1177/0284185117725367 28766979

[31] Tsuchiya N Doai M Usuda K Uramoto H Tonami H. Non-small cell lung cancer: Whole-lesion histogram analysis of the apparent diffusion coefficient for assessment of tumor grade, lymphovascular invasion and pleural invasion. PloS One (2017) 12(2):e0172433. doi: 10.1371/journal.pone.0172433 28207858 PMC5313135

[32] Liang P Xu C Tan F. Prediction of the World Health Organization Grade of rectal neuroendocrine tumors based on CT histogram analysis. Cancer Med (2021) 10(2):595–604. doi: 10.1002/cam4.3628 33263225 PMC7877354

[33] Zhu L Bian H Yang L. (18) Fluorodeoxyglucose-positron emission tomography/computed tomography features of suspected solitary pulmonary lesions in breast cancer patients following previous curative treatment. Thorac Cancer (2019) 10(5):1086–95. doi: 10.1111/1759-7714.13049 PMC6500976 30900387

[34] Song L Yin J. Application of texture analysis based on sagittal fat-suppression and oblique axial T2-weighted magnetic resonance imaging to identify lymph node invasion status of rectal cancer. Front Oncol (2020) 10:1364. doi: 10.3389/fonc.2020.01364 32850437 PMC7426518

[35] Zheng X Guo W Dong J Qian L. Prediction of early response to concurrent chemoradiotherapy in cervical cancer: Value of multi-parameter MRI combined with clinical prognostic factors. Magn Reson Imaging (2020) 72:159–66. doi: 10.1016/j.mri.2020.06.014 32621877

[36] Nerad E Delli Pizzi A Lambregts DMJ. The Apparent Diffusion Coefficient (ADC) is a useful biomarker in predicting metastatic colon cancer using the ADC-value of the primary tumor. PloS One (2019) 14(2):e0211830. doi: 10.1371/journal.pone.0211830 30721268 PMC6363286

[37] Peng Y Tang H Hu X. Rectal cancer invasiveness: whole-lesion diffusion-weighted imaging (DWI) histogram analysis by comparison of reduced field-of-view and conventional DWI techniques. Sci Rep (2019) 9(1):18760. doi: 10.1038/s41598-019-55059-0 31822707 PMC6904447

